# Effects of brain activity, morning salivary cortisol, and emotion regulation on cognitive impairment in elderly people

**DOI:** 10.1097/MD.0000000000016114

**Published:** 2019-06-28

**Authors:** Watchara Sroykham, Yodchanan Wongsawat

**Affiliations:** Department of Biomedical Engineering, Faculty of Engineering, Mahidol University, Thailand.

**Keywords:** brain activity, cognitive impairment, cortisol, elderly, emotion

## Abstract

**Objective:**

Cognitive impairment subjects exhibit high cortisol levels that are associated with low brain activity, but negative emotions with high cortisol are associated with high brain activity and reduced cognition. Emotion regulation, glucocorticoid hormones, and brain activity all interact with cognitive impairment. Therefore, we aimed to investigate cognitive impairment differences related to sex, morning salivary cortisol, emotion regulation, and brain activity in elderly people.

**Methods:**

A total of 64 participants (19 males and 45 females) were tested by the Montreal cognitive assessment. Next, morning saliva was collected from each participant and analyzed by enzyme-linked immunosorbent assay, and the brain activity of the participants was subsequently recorded. Finally, emotion regulation was assessed via the Brunel mood scale questionnaire.

**Results:**

The results revealed that attention was significantly lower in elderly females than in elderly males. Depression and vigor were significantly higher in elderly females than in elderly males. Brain activity of the slow (delta and theta) and fast (beta and high beta) waves was significantly higher in elderly females than in elderly males. Moreover, attention was negatively correlated with the theta wave, whereas delayed recall was positively correlated with the theta wave and salivary cortisol. Depression was positively correlated with the high beta wave and language skill, whereas the high beta wave was negatively correlated with visuoconstructional skill.

**Conclusion:**

The brain activity, emotion, and cortisol were influenced by cognitive impairments, although the relation of brain activity with glucocorticoid hormones remains inconclusive. This finding may be useful to the brain aging process, promote healthy brain aging, and prevent neurodegenerative conditions.

## 1 Introduction

Cognitive impairment is a normal process of aging. The most common type of cognitive impairment among the elderly population is mild cognitive impairment (MCI), which is the intermediate stage between normal brain function and full dementia.^[1]^ MCI and dementia are related to the hippocampus region of the brain and have been associated with elevated cortisol levels.^[2]^

Cortisol regulates metabolism, blood glucose levels, immune responses, anti-inflammatory actions, blood pressure, and emotion regulation. Cortisol is a glucocorticoid hormone that is synthesized and secreted by the cortex of adrenal glands. The hypothalamus releases a corticotrophin-releasing hormone and arginine vasopressin into hypothalamic-pituitary portal capillaries, which stimulates adrenocorticotropic hormone secretion, thus regulating the production of cortisol. Basal cortisol elevation causes damage to the hippocampus and impairs hippocampus-dependent learning and memory. Chronic high cortisol causes functional atrophy of the hypothalamic-pituitary-adrenal axis (HPA), the hippocampus, the amygdala, and the frontal lobe in the brain.^[3]^ Previous studies have shown that higher HPA activity increases plasma cortisol levels, which is associated with more rapid disease progression in subjects with Alzheimer-type dementia (AD).^[4]^ Increased saliva cortisol is associated with nonamnestic or multidomain MCI, and cerebrospinal fluid (CSF) cortisol concentrations were increased in subjects with AD dementia or MCI-AD.^[5]^ Moreover, stress also activates the HPA axis via a neural connection to the paraventricular nucleus from many parts of the brain.^[6]^ Increasing cortisol levels are related to a number of psychosocial factors,^[7]^ major depression, and stress,^[8]^ and emotion regulation influences a variety of cognitive processes.^[9]^

Cortisol also affects brain activity. Cortisol increased the relative right frontal activity and reduces approach motivation,^[10]^ was significantly associated with midfrontal delta–beta coupling and correlated with slow wave (delta) and fast wave (beta) activity,^[11]^ and was associated with anxiety and behavioral inhibition.^[12]^ Stress activates the HPA axis activity and brain activity, increasing frontal activity asymmetry.^[13]^ Furthermore, MCI subjects with cerebrovascular damage are associated with increased delta power and decreased alpha2 power. Moderate hippocampal atrophy exhibited the highest increase in alpha2 and alpha3 power, and an increased theta/gamma ratio was highly associated with amygdala atrophy. In addition, the alpha3/alpha2 ratio was strongly associated with hippocampal atrophy.^[14]^

Interestingly, cognitive impairment subjects exhibited high cortisol levels that were associated with low brain activity, but negative emotions with high cortisol were associated with high brain activity. Emotion regulation, glucocorticoid hormones, and brain activity all interacted with cognitive impairment. However, the relationship among cortisol, brain activity, and emotion regulation in cognitive impairment remains inconclusive. Therefore, we aimed to investigate cognitive impairment, including differences related to sex, morning salivary cortisol, emotion regulation, and brain activity in elderly people.

## 2 Participants and methods

### 2.1 Participants

Participants were recruited from the healthy brain project of Mahidol University but were excluded from this study if they were younger than 50 years or had a history of epilepsy or migraines, resting heart rate >120 beats per minute, resting systolic blood pressure >180 mmHg, resting diastolic blood pressure >100 mmHg, or blood oxygen saturation <90%. All participants (male = 19, female = 45) were healthy, older than 50 years and did not have epilepsy or migraines; all participants provided written informed consent before participation.

### 2.2 Procedure

In the morning between 08:00 and 10:00 am, the cognitive impairment of all participants was tested by the Montreal cognitive assessment (MoCA). Next, all participants rinsed their mouth with water and spit into a saliva container to collect approximately 5 mL of saliva. The saliva samples were collected in approximately 15-minute intervals and kept at 4°C. Then, an electroencephalography (EEG) cap was placed on each participant's head according to the international 10–20 system. Brain activity was recorded for 5 minutes in the eyes-open condition using a Discovery 24E (BrainMaster) system. Finally, emotion regulation was assessed via the Brunel mood scale questionnaire (BRUMS). This study was approved by the Medical Ethical Committee of Mahidol University under approval number Mahidol University Institutional Review Board (MU-IRB) 2013/042.1004.

### 2.3 Measurements

**Figure 1:**
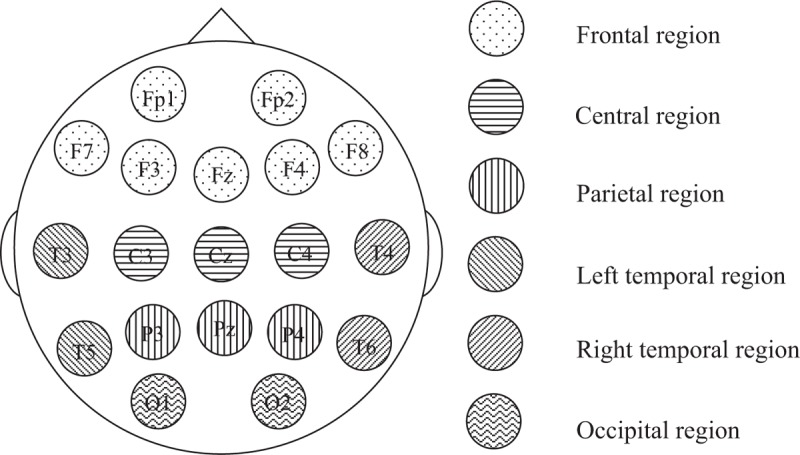
Brain regions for the quantitative electroencephalogram analysis.

Cognitive impairment was assessed via MoCA to measure visuospatial skills, naming, attention, language, abstraction, delayed recall, and orientation. MoCA is scored out of 30, with a score <26 indicating mind cognitive impairment. The morning salivary cortisol levels (nmol/L) were analyzed by enzyme-linked immunosorbent assay at iPathMedlabs Pty Ltd., Australia. The reference rang of morning salivary cortisol was 6.0 to 42.0 nmol/L. The brain signals were recorded by 24-bit analog-to-digital converters at a 256-Hz sampling rate and a bandwidth of 0.00 Hz to 80.00 Hz for 5 minutes in the eyes-open condition. The free artifacts of 1 minute of EEG from the 5 minutes had a test–retest reliability >0.90 and a split-half reliability >0.95. The fast Fourier transform (FFT) absolute power of these free artifacts of the brain signals was computed and compared with the NeuroGuide normative database (*z* score FFT absolute power, 625 individuals, 2 months–82.6 years). The bands of brain activity included delta wave (0.5–4 Hz), theta wave (4–8 Hz), alpha wave (8–12 Hz), beta wave (12–25 Hz), and high beta wave (25–30 Hz). The *z* score of FFT absolute power was averaged into 6 regions: the frontal region (FP1, F3, F7, Fz, FP2, F4, and F8), the central region (C3, Cz, and C4), the parietal region (P3, Pz, and P4), the left temporal region (T3 and T5), the right temporal region (T4 and T6), and the occipital region (O1 and O2) (Fig. 1). Emotion regulation was assessed via the BRUMS, which was developed to investigate mood states. The BRUMS is a 24-item mood scale that measures 6 identifiable affective states (tension, depression, anger, vigor, fatigue, and confusion). Each item is scored on a range of 0 to 4 (0 indicates “not at all,” 1 indicates “a little,” 2 indicates “moderately,” 3 indicates “quite a bit,” and 4 indicates “extremely”).

### 2.4 Statistical analysis

Statistical analyses were performed with PASW Statistics 18.0 (IBM Corporation, Armonk, NY). The datasets were tested for normality using the Shapiro-Wilk test. The average, standard deviation (SD), and standard error of the mean (SEM) were calculated in males and females. The averages of the results were compared between males and females using independent-samples *t* tests for normal datasets and independent-samples Mann–Whitney *U* tests for non-normal datasets with 95% confidence intervals. The correlations between cognitive impairment, morning salivary cortisol, emotion regulation, and brain activity were analyzed by Spearman correlation with 95% confidence intervals.

## 3 Results

### 3.1 Cognitive impairment

The study population included 64 participants (19 males and 45 females). The cognitive impairment of participants assessed via the MoCA that showed overall of cognitive performance was higher in males than in females. The attention was significantly different between sexes (*P* = .008). No significant statistical difference was found in age (*P* = 0.702), MoCA score (*P* = .090), visuoconstructional skill (*P* = .066), naming (*P* = .134), language (*P* = .784), abstraction (*P* = .934), delayed recall (*P* = .471), and orientation (*P* = 0.463) between sexes (Table 1).

**Table 1:**
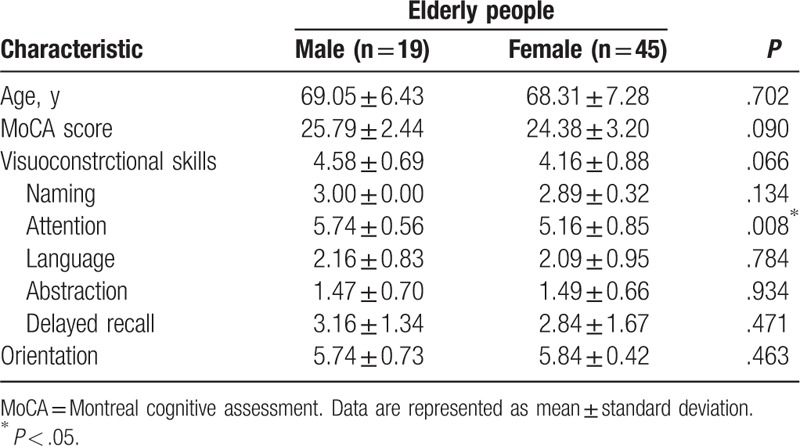
Age and cognitive impairment of elderly male and elderly female.

### 3.2 Morning salivary cortisol

**Figure 2:**
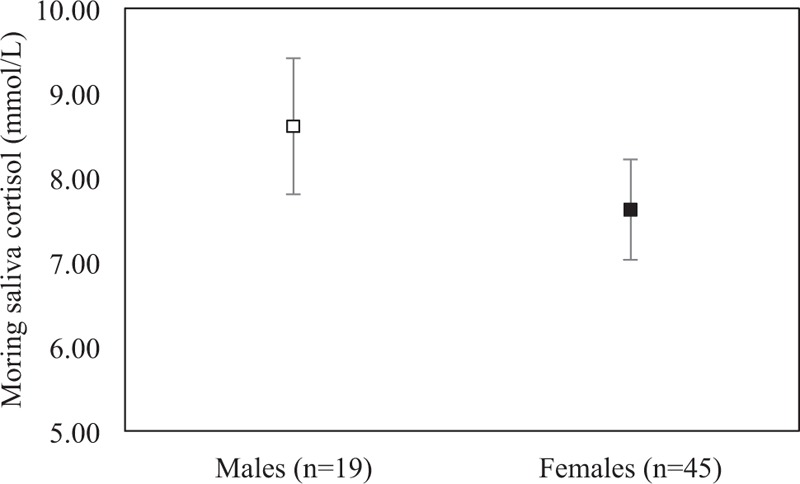
The mean morning salivary cortisol concentrations (mmol/L) in elderly males (n = 19) and elderly females (n = 45). Error bars represent standard error of the mean.

The mean morning salivary cortisol levels were higher in males 8.61 (SEM = 0.80) mmol/L than in females 7.62 (SEM = 0.59) mmol/L, which was not significantly different between sexes (*P* = .351) (Fig. 2).

### 3.3 Emotion regulation

**Figure 3:**
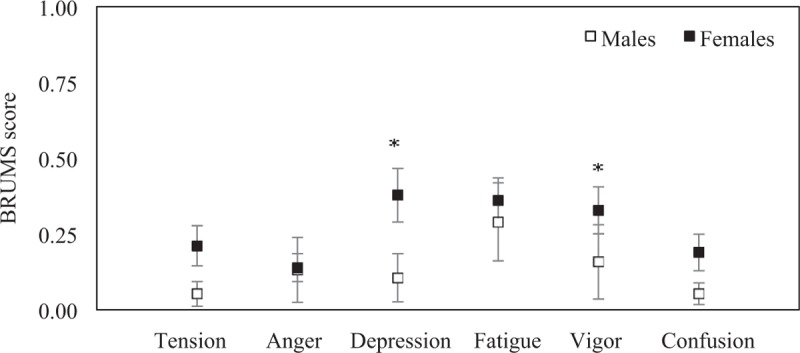
Emotion regulation of participants assessed via the Brunel mood scale questionnaire. The mean of tension, anger, depression, fatigue, vigor, and confusion in elderly males (n = 19) and elderly females (n45). Error bars represent standard error of the mean (^∗^*P* < .05).

The emotion regulation of participants assessed via the BRUMS that showed overall of emotion regulation was higher in females than males. The mean tension was 0.05 (SEM = 0.04) in males and 0.21 (SEM = 0.07) in females. The mean anger was 0.13 (SEM = 0.11) in males and 0.14 (SEM = 0.05) in females. The mean depression was 0.11 (SEM = 0.08) in males and 0.38 (SEM = 0.09) in females. The mean fatigue was 0.29 (SEM = 0.13) in males and 0.36 (SEM = 0.07) in females. The mean vigor was 0.16 (SEM = 0.12) in males and 0.33 (SEM = 0.08) in females. The mean confusion was 0.05 (SEM = 0.04) in males and 0.19 (SEM = 0.06) in females. The depression and vigor were significantly different between sexes (*P* = .023 and *P* = .022). No significant statistical difference was found in tension (*P* = 0.109), anger (*P* = .263), fatigue (*P* = .353), and confusion (*P* = 0.161) between sexes (Fig. 3).

### 3.4 Brain activity

**Figure 4:**
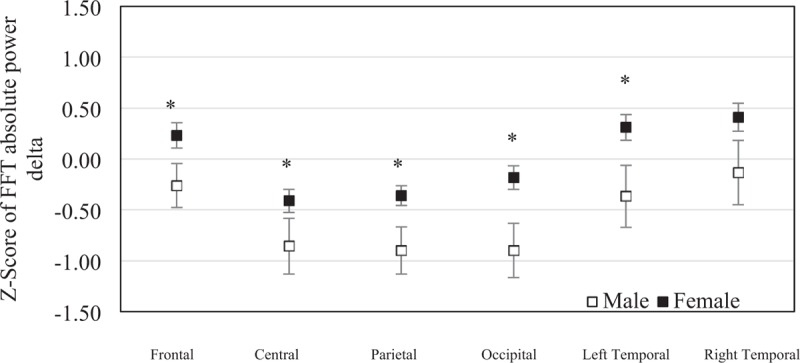
The mean of *z* scores of the fast Fourier transform absolute delta powers at each brain region were averaged in elderly males (n = 19) and elderly females (n = 45). Error bars represent standard error of the mean (^∗^*P* < .05).

The mean of *z* score of the FFT absolute delta power was −0.261 (SEM = 0.22) in males and 0.233 (SEM = 0.12) in females at frontal region, −0.857 (SEM = 0.28) in males and −0.411 (SEM = 0.11) in females at central region, −0.900 (SEM = 0.23) in males and −0.361 (SEM = 0.10) in females at parietal region, −0.898 (SEM = 0.27) in males and −0.183 (SEM = 0.12) in females at occipital region, −0.367 (SEM = 0.31) in males and 0.311 (SEM = 0.13) in females at left temporal region, and −0.134 (SEM = 0.32) in males and 0.410 (SEM = 0.14) in females at right temporal region. The central, parietal, occipital, and left temporal regions were significantly different between sexes (*P* = .034, *P* = .019, *P* = .009, and *P* = .018). No significant statistical difference was found in frontal region (*P* = .059) and right temporal region (*P* = .072) between sexes (Fig. 4).

**Figure 5:**
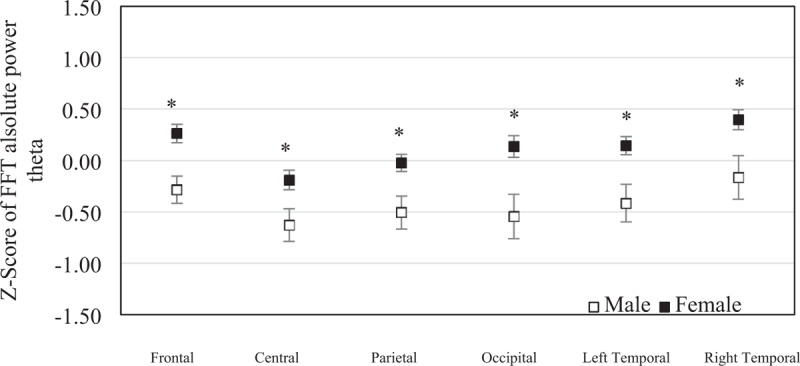
The mean of *z* scores of the fast Fourier transform absolute theta powers at each brain region were averaged in elderly males (n = 19) and elderly females (n = 45). Error bars represent standard error of the mean (^∗^*P* < .05).

The mean of *z* score of the FFT absolute theta power was −0.285 (SEM = 0.13) in males and 0.262 (SEM = 0.09) in females at frontal region, −0.630 (SEM = 0.16) in males and −0.190 (SEM = 0.10) in females at central region, −0.506 (SEM = 0.16) in males and −0.024 (SEM = 0.09) in females at parietal region, −0.544 (SEM = 0.22) in males and 0.137 (SEM = 0.12) in females at occipital region, −0.416 (SEM = 0.18) in males and 0.143 (SEM = 0.09) in females at left temporal region, and −0.164 (SEM = 0.21) in males and 0.396 (SEM = 0.10) in females at right temporal region. The frontal, central, parietal, occipital, left temporal, and right temporal regions were significantly different between sexes (*P* = .002, *P* = .033, *P* = .019, *P* = .008, *P* = .007 and *P* = .013) (Fig. 5).

**Figure 6:**
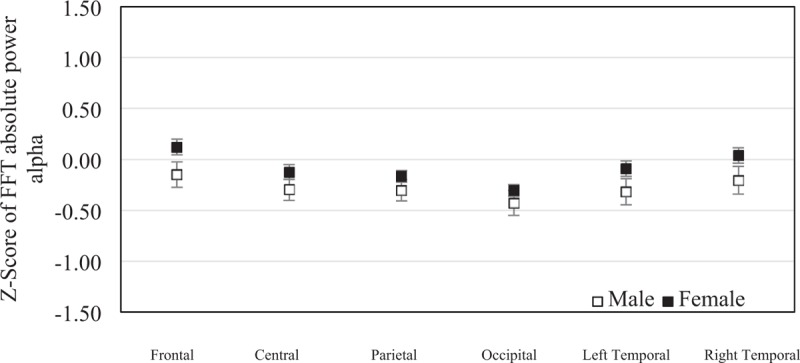
The mean of *z* scores of the fast Fourier transform absolute alpha powers at each brain region were averaged in elderly males (n = 19) and elderly females (n = 45). Error bars represent standard error of the mean (^∗^*P* < .05).

The mean of *z* score of the FFT absolute alpha power was −0.149 (SEM = 0.13) in males and 0.120 (SEM = 0.08) in females at frontal region, −0.298 (SEM = 0.11) in males and −0.125 (SEM = 0.07) in females at central region, −0.305 (SEM = 0.10) in males and −0.167 (SEM = 0.06) in females at parietal region, −0.429 (SEM = 0.12) in males and −0.306 (SEM = 0.06) in females at occipital region, −0.319 (SEM = 0.13) in males and −0.091 (SEM = 0.08) in females at left temporal region, and −0.207 (SEM = 0.14) in males and 0.037 (SEM = 0.07) in females at right temporal region. No significant statistical difference was found in frontal region (*P* = .063), central region (*P* = .220), parietal region (*P* = .243), occipital region (*P* = .280), left temporal region (*P* = .121), and right temporal region (*P* = 0.77) between sexes (Fig. 6).

**Figure 7:**
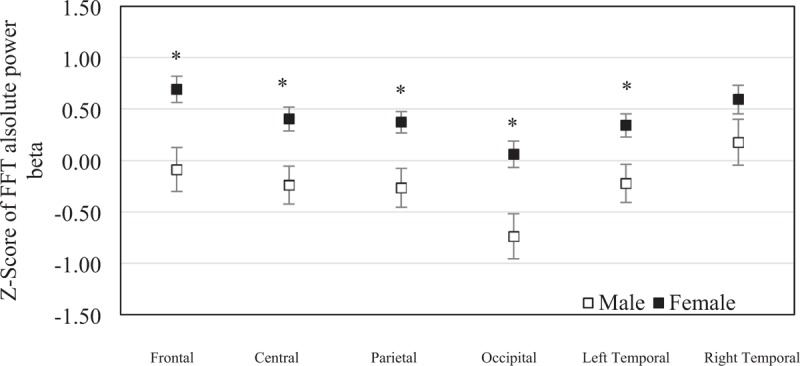
The mean of *z* scores of the fast Fourier transform absolute beta power at each brain region were averaged in elderly males (n = 19) and elderly females (n = 45). Error bars represent standard error of the mean (^∗^*P* < .05).

The mean of *z* score of the FFT absolute beta power was −0.088 (SEM = 0.21) in males and 0.692 (SEM = 0.13) in females at frontal region, −0.238 (SEM = 0.19) in males and 0.405 (SEM = 0.12) in females at central region, −0.266 (SEM = 0.19) in males and 0.372 (SEM = 0.10) in females at parietal region, −0.738 (SEM = 0.22) in males and 0.060 (SEM = 0.13) in females at occipital region, −0.224 (SEM = 0.18) in males and 0.342 (SEM = 0.11) in females at left temporal region, and 0.176 (SEM = 0.22) in males and 0.594 (SEM = 0.14) in females at right temporal region. The frontal, central, parietal, occipital, and left temporal regions were significantly different between sexes (*P* = .003, *P* = .009, *P* = .003, *P* = .003 and *P* = .017). No significant statistical difference was found in right temporal region (*P* = .111) between sexes (Fig. 7).

**Figure 8:**
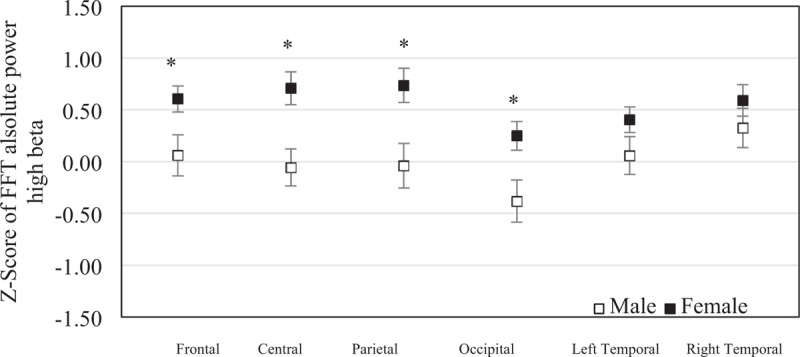
The mean of *z* scores of the fast Fourier transform absolute high beta powers at each brain region were averaged in elderly males (n = 19) and elderly females (n = 45). Error bars represent standard error of the mean (^∗^*P* < .05).

The mean of *z* score of the FFT absolute high beta power was 0.060 (SEM = 0.20) in males and 0.605 (SEM = 0.13) in females at frontal region, −0.058 (SEM = 0.18) in males and 0.707 (SEM = 0.16) in females at central region, −0.040 (SEM = 0.21) in males and 0.737 (SEM = 0.17) in females at parietal region, −0.382 (SEM = 0.20) in males and 0.248 (SEM = 0.14) in females at occipital region, 0.058 (SEM = 0.18) in males and 0.405 (SEM = 0.12) in females at left temporal region, and 0.323 (SEM = 0.19) in males and 0.591 (SEM = 0.15) in females at right temporal region. The frontal, central, parietal and occipital regions were significantly different between sexes (*P* = .021, *P* = .008, *P* = .007, and *P* = .012). No significant statistical difference was found in left temporal region (*P* = .169) and right temporal regions (*P* = .423) between sexes (Fig. 8).

### 3.5 Correlations between cognitive impairment, morning salivary cortisol, emotion regulation and brain activity

The correlations between cognitive impairment, morning salivary cortisol, emotion regulation, and brain activity of participants revealed that attention was negatively correlated with the *z* score of the FFT absolute theta power (Spearman rho (64) = −0.271, *P* = .030), whereas delayed recall was positively correlated with the *z* score of the FFT absolute theta power (Spearman rho (64) = 0.252, *P* = .045). Moreover, delayed recall was positively correlated with salivary cortisol (Spearman rho (64) = 0.294, *P* = .018). The *z* score of the FFT absolute high beta power was negatively correlated with visuoconstrctional skills (Spearman rho (64) = −0.362, *P* = .003) but was positively correlated with depression (Spearman rho (64) = 0.266, *P* = .033). Moreover, depression was positively correlated with language (Spearman rho (64) = 0.256, *P* = .041). Notably, morning salivary cortisol was not correlated with emotion regulation or brain activity (Table 2).

**Table 2:**
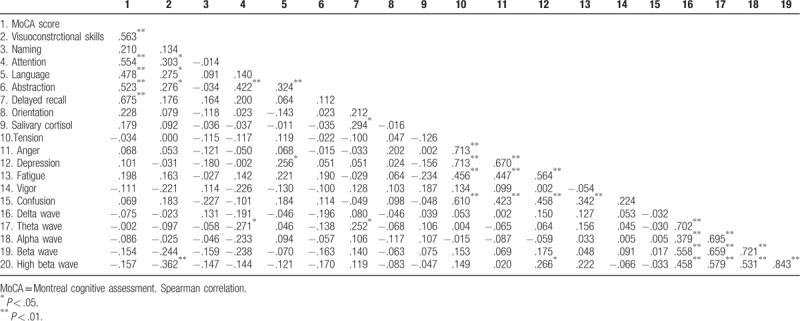
Correlation between attention impairment, morning salivary cortisol, emotion regulation and brain activity.

## 4 Discussion

The present study aimed to investigate cognitive impairment including differences related to sex, morning salivary cortisol, emotion regulation, and brain activity in elderly people. The MoCA is scored out of 30, with a score <26 indicating mind cognitive impairment. The results showed that the cognitive performance of visuoconstructional skills, naming, language, abstraction, delayed recall, orientation, and MoCA score was not different in elderly males and elderly females. Interestingly, the results revealed that attention in elderly females was significantly lower than in elderly males. The results revealed that the depression and vigor were significantly higher in elderly females than in elderly males. It should be noted that the brain signals of participants were computed and compared with the control group using the NeuroGuide normative database (*z* score FFT absolute power collected from 625 individuals aged from 2 months to 82.6 years) and the *z* score of FFT absolute power was averaged into 6 regions: the frontal region, the central region, the parietal region, the left temporal region, the right temporal region, and the occipital region. The results revealed that brain activity of slow waves (delta and theta) and fast waves (beta and high beta) was significantly higher in elderly females than in elderly males. However, morning salivary cortisol was not different between sexes. Moreover, these results revealed that the effect of attention was negatively correlated with theta wave but was not correlated with emotion regulation or morning salivary cortisol, whereas delayed recall was positively correlated with theta wave and morning salivary cortisol. The effect of depression was positively correlated with high beta wave and language, whereas visocontructional skills were negatively correlated with high beta wave. Notably, morning salivary cortisol was not correlated with emotion regulation or brain activity.

MCI is the intermediate stage between normal brain function and dementia, and hippocampal volume decreases as MCI progresses. MCI subjects display impaired attentional processing, working memory capacity, and semantic language.^[15]^ MCI subjects have been shown to exhibit increased delta power and decreased alpha power. Moreover, increased theta rhythms have been consistently associated with amygdalohippocampal complex atrophy and memory deficits and are a major risk for the development of Alzheimer disease and MCI.^[16]^ Theta power has been correlated with inattention and executive problems,^[17]^ whereas beta power is decreased in low-performing elderly subjects with deficits in sustaining attentional processes.^[18]^ This result revealed that low attention was associated with high slow wave brain activity (delta and theta waves) and was negatively correlated with theta waves. This finding indicates that the effect of brain activity was strong and consistent with attentional cognitive impairment in elderly. Moreover, delayed recall was associated with cortisol level and theta wave. In previous studies, cortisol was associated with memory decline in elderly people. Therefore, basal cortisol elevation may cause hippocampal damage and impair hippocampus-dependent learning and memory in humans. Many studies have shown that increased salivary cortisol, plasma cortisol, and CSF cortisol in subjects are associated with MCI or dementia. Different subtypes of MCI have been associated with saliva cortisol; for example, increased salivary cortisol levels have been shown in nonamnestic MCI and multidomain MCI, whereas normal levels are found in amnestic MCI. Furthermore, some studies showed that cortisol concentration was not associated with MCI.^[19]^ Our results revealed that delayed recall was correlated with morning salivary cortisol. However, attention and delayed recall were not strongly correlated with brain activity and cortisol levels. Other cognitive function test may strongly explain the relationship between memory and cortisol regulation. Chronic high cortisol may damage the hippocampus, induce MCI, and reduce the brain function of elderly. Chronic high cortisol is a major risk factor associated with the development of dementia.

Emotion recognition and emotion processing appear with dysfunction during aging or dementia. Alzheimer disease patients exhibit worse emotions of anger, sadness, and fear than MCI patients and healthy individuals.^[20]^ This could be explained by the hypothesis that hippocampal and amygdala atrophy in MCI lead to deficits in emotional regulation and brain circuitry.^[21]^ However, personality traits may influence cognitive impairment, and high cortisol has been associated with high extraversion and low openness.^[22]^ The results revealed low attention was high depression and vigor in elderly female. Interestingly, depression was positively correlated with beta wave and language. The EEG at the right frontal lobe was activated by stress and was associated with increased cortisol awakening responses.^[23]^ Stress activates acute high cortisol levels, and chronic stress also increases the risk for depression and mental illness. Stress activates the HPA to secrete cortisol, which is associated with damage to the hippocampus and other brain regions. Chronic cortisol is a cause of hippocampal atrophy and brain activity asymmetry. Moreover, cortisol administration significantly changed frontal asymmetry and was associated with a significant increase in the correlation between delta and beta activity in the midfrontal lobe. This finding indicates that the effect of depression was activated by brain activity. However, Spearman’ rho values do not only reach 0.266, which is generally interpreted as a weak to moderate relationship.

There are some limitations of the present study. Healthy elderly and young people were not included in this study and compared with the cognitive impairment in elderly people. This study investigated only morning cortisol levels. It is circadian hormone that typically rises in the early morning, decreases throughout the rest of the day, and is the lowest around midnight. Moreover, estrone, estradiol, progesterone, follicle-stimulating hormone, and luteinizing hormone levels have been shown to be correlated with EEG and depression in elderly females.^[24]^ This study did not test whether these hormones were influenced by brain activity, emotion, or cognitive impairment. This study used only the absolute power of EEG, although EEG coherence is another parameter used to characterize patients with attention deficit disorders.^[25]^ According to a future study, longitudinal study in an aging population with physiological, psychological, and behavioral changes must be included in the neuroendocrine study.

In summary, the present study represents the first attempt to identify the effects of brain activity, cortisol hormone, and emotion regulation on cognitive impairment. The brain activity, emotion, and cortisol were influenced by cognitive impairments, although the relation of brain activity with glucocorticoid hormones remains inconclusive. This finding may be useful to the brain aging process, promote healthy brain aging, and prevent neurodegenerative conditions. Future studies are needed to clarify the effects of hormone imbalance on brain activity and emotion regulation in cognitively impaired elderly by evaluating low and high hormone imbalance groups. Moreover, cognitive function tasks and other brain activity analyses were evaluated in this study.

## Acknowledgment

This project was supported in part by Mahidol University and the Siam Cement Group (SCG). The success of this research is attributed to the extensive support, valuable advice, and guidance from Asst. Prof. Sirintorn Chansirikarnjana, MD, Faculty of Medicine, Ramathibodi Hospital, Assoc. Prof. Sureeporn Punpuing, PhD, Institute for Population and Social Research, and Asst. Prof. Jatuporn Wongsathikun, PhD, Faculty of Physical Therapy, Mahidol University.

## Author contributions

**Writing – original draft:** Watchara Sroykham.

**Writing – organizing draft, review & editing:** Yodchanan Wongsawat.
